# Supplementary figures and images for: Hypoxia-Induced Adaptations of Embryonic Fibroblasts: Implications for Developmental Processes
Source: Biology (Basel). 2024 Aug 8;13(8):598. doi: 10.3390/biology13080598 (PMC11351757; doi:10.3390/biology13080598)

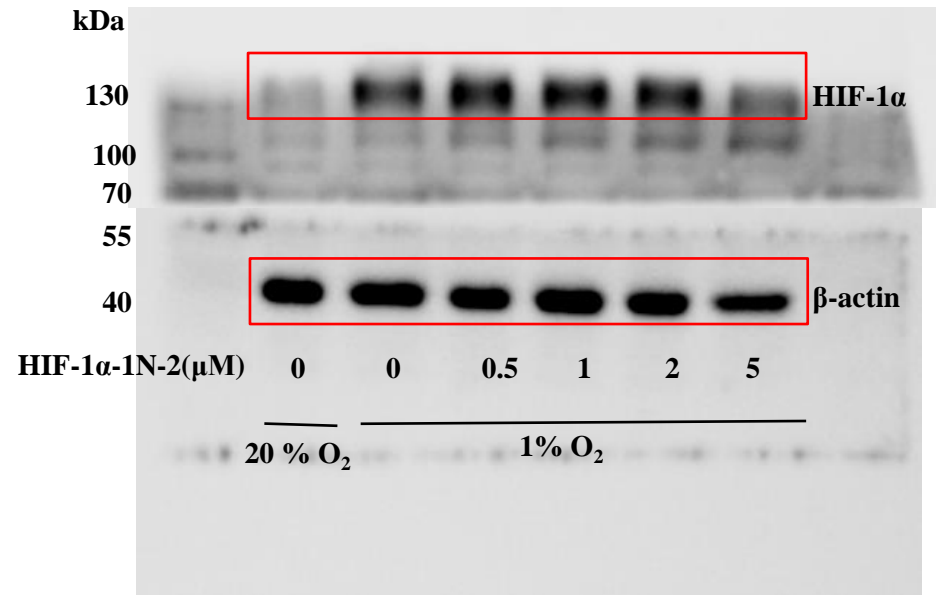

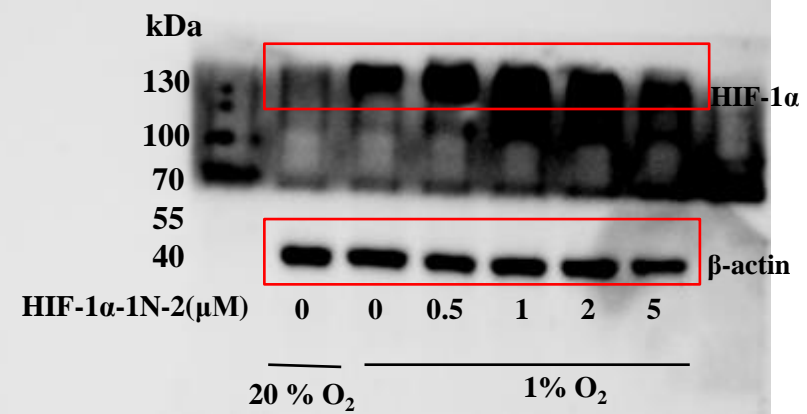

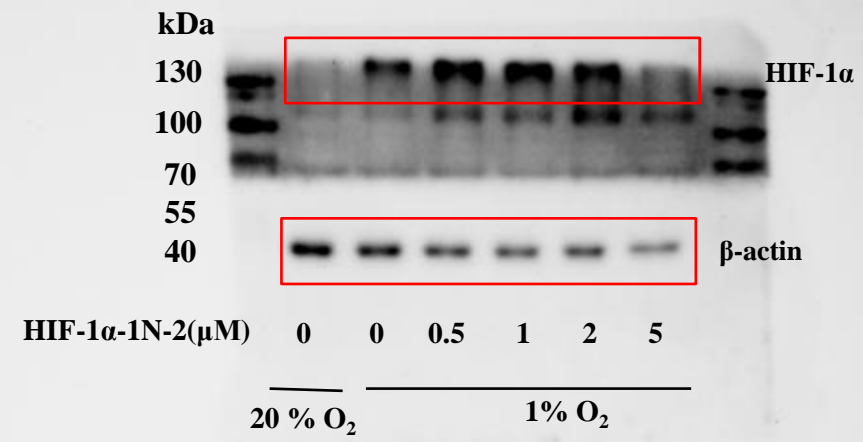

Supplement: Supplementary file 1 [file biology-13-00598-s001.zip › Figure S3 uncropped gels.pdf]
